# Dependence of far-red light on red and green light at increasing growth of lettuce

**DOI:** 10.1371/journal.pone.0313084

**Published:** 2024-11-15

**Authors:** Nathan Kelly, Erik S. Runkle

**Affiliations:** Department of Horticulture, Michigan State University, East Lansing, Michigan, United States of America; Wageningen University, NETHERLANDS, KINGDOM OF THE

## Abstract

Despite being outside of the traditionally defined photosynthetically active radiation (PAR) waveband (400–700 nm), far-red (FR; 700–799 nm) light can increase photosynthesis and induce shade-avoidance responses, which increases light interception and thus, whole-plant growth. However, it is unclear how the promotion of growth from FR light depends on PAR wavebands and specifically how the substitution of red light (600–699 nm) with green light (500–599 nm) influences the efficacy of FR light on increasing shoot biomass accumulation. To determine this, we grew red- and green-leaf lettuce (*Lactuca sativa*) at a fixed total photon flux density (PFD) with 12 different fractions of red, green, and FR light and the same PFD of blue (400–499 nm) light. We postulated that decreasing the red:FR by substituting FR light for green light, red light, or both would increase shoot fresh mass (FM) until a fraction beyond which growth (but not leaf area) would begin to decrease. Indeed, the substitution of red with FR light increased the leaf area of both cultivars, but FM was greatest under an FR fraction [FR/(R+FR)] of approximately 0.25. Under the greatest FR PFD, FM was similar to lettuce grown without FR light, despite having greater leaf surface area for light interception. Green light had less of an effect on leaf expansion and FM than FR light, and plant diameter and leaf area of red-leaf ‘Rouxai’ were the greatest when green light fully replaced red light at the highest FR PFD. We conclude that under a modest light intensity and blue PFD, a spectrum that includes up to 25% of far-red photons can increase leaf area and biomass accumulation. While leaf area may continue to increase at higher far-red fractions, fresh mass does not, and plant quality begins to deteriorate.

## Introduction

Light-emitting diodes (LEDs) are increasingly being used in plant growth facilities because of their high photosynthetic photon efficacies, long operating lifetime, low heat emission, and the possibility to fine-tune the light spectrum [1]. The potential of customizing the light spectrum enables researchers and commercial growers to regulate the growth, morphology, and quality attributes of their crops. For example, a relatively high fraction of blue (B; 400–499 nm) light produces more compact plants with higher concentrations of phenolic compounds compared to a spectrum with less blue light [2, 3]. Red (R; 600–699 nm) and blue LEDs efficiently drive plant photosynthesis and convert electricity to emitted photons, which is quantified by the micromoles of photons output per joule (μmol∙J^−1^) of input power [1]. White LEDs are commonly incorporated into LED fixtures because of their low cost and broad spectrum, but white LEDs (and to a lesser degree, R LEDs) emit a small percentage of far-red (FR; 700–799) light. FR light is outside of the traditionally defined photosynthetically active radiation (PAR) waveband of 400−700 nm but it increases leaf expansion, light interception, and plant photosynthesis [4–6]. In addition, some FR LEDs are even more electrically effective than many red LEDs [1]. Thus, greater adoption of FR LEDs in plant lighting fixtures can potentially increase growth per unit of electricity consumed.

FR light has extensive effects on plant morphology and growth because it is absorbed by plant photoreceptors, particularly phytochrome, and it directly enhances photosynthesis.

Phytochrome (phy) is a group of photoreceptors that primarily absorbs red and FR light. Each type of phytochrome exists in two forms (Pr and Pfr) that interconvert, primarily depending on the proportion of red and FR light in the spectrum. The phytochrome photoequilibrium (PPE), which is the ratio of Pfr to Pr+Pfr, can be estimated for plants grown under a specific light spectrum [7, 8]. The PPE can be refined to incorporate spectral distortions that occur inside leaves caused by photon scattering and absorption by proteins, which is referred to as the internal PPE (iPPE) [7]. Phytochrome B is converted to its inactive form (Pr) when it is exposed to FR light [(a low red to far-red light ratio (R:FR)] [9]. The Pr form of phy then dissociates from phytochrome-interacting factors 4 and 5 (PIF4, PIF5) to promote the expression of genes involved in shade-avoidance responses, such as cell elongation [10, 11]. Shade-avoidance responses caused by FR light’s interaction with phytochrome can lead to an increase in leaf area and canopy size, indirectly increasing photosynthesis from greater light capture, but the magnitude of response can depend on other light wavebands, total photon flux density, and plant density [12–15]. For example, adding FR light during the day or at the end-of-the day increased leaf area, radiation-use efficiency, and total dry mass of indoor-grown lettuce (*Lactuca sativa*) [16].

FR light also enhances plant photochemistry and photosynthesis when combined with PAR wavebands, such as red light, by preferentially exciting photosystem I (PSI) [5, 17]. This phenomenon was first described as the Emerson Enhancement Effect, which states that photosynthetic rates are greater when light of 670–680 nm and >680 nm are applied together compared with the sum of the two wavebands applied separately [18, 19]. More recently, Zhen et al. [20] demonstrated that adding red and FR light at wavelengths from 686 to 703 nm to red+blue light, or a simulated solar spectrum, progressively increased the quantum yield of photosystem II (PSII) and photosynthetic rates by exciting PSI and restoring excitation balance between the two photosystems. Additionally, FR light of 721 to 731 nm similarly increased the quantum yield of PSII as 703-nm light [20]. These studies have shown that specific wavelengths of FR light can independently drive plant photosynthesis, or synergistically when added to PAR wavebands, and thus increase subsequent plant growth.

These morphological and photosynthetic effects of FR light lead to vast changes in plant architecture and growth. In addition to FR light, the R:FR of a light spectrum regulates plant growth, morphology, and quality attributes such as leaf coloration, leaf thickness (texture), and nutritional quality. For example, decreasing the R:FR by adding FR light to a broad-waveband (white light) spectrum decreased anthocyanin concentration and increased shoot fresh mass (FM), stem length, leaf length, and leaf width of lettuce compared to the white-light control treatment [21]. Additionally, adding FR light to red+blue light increased plant height, leaf area, and dry mass of ornamental seedlings [4, 22]. Moreover, FR light added to red+blue light increased total plant phenolic [23] and soluble sugar content, but decreased chlorophyll content, as the leaf area of lettuce increased [16]. The magnitude of FR light responses can be influenced by other wavebands or the background photon flux density (PFD; μmol∙m^−2^∙s^−1^). For example, adding FR light to a red+blue spectrum increased lettuce shoot FM and leaf length, but the effect was more pronounced under a high blue:red or low PFD than a lower blue:red or higher total photon flux density (TPFD; 300–799 nm), respectively [24]. Finally, substituting blue light with FR light increased leaf expansion and FM of lettuce [25].

Green light (500–599 nm), on the other hand, is thought to have minor effects on plant growth due to its weaker absorption by chlorophyll and reflectance by plant leaves [17, 26]. However, green light passes through leaf tissue and thus penetrates deeper into plant leaves and canopy than red or blue light, increasing whole-plant photosynthesis [27, 28]. Despite the misconception that green light is less effective at increasing biomass, FM of lettuce was similar or slightly increased at a higher photosynthetic PFD (PPFD) when red+blue light was partially replaced with up to ≈10% green light [29]. In another study, replacing red light with an equal PPFD of green light increased lettuce FM and leaf area [30]. Finally, similar to FR light, green light induces some shade-avoidance-like responses (e.g., increased leaf size) when it replaces blue light in a red+blue spectrum [25]. However, not all studies have reported positive effects of green light among species studied. For example, as the percentage of green light increased from 1 to 51%, tomato dry mass and leaf area increased, but there was no effect on lettuce or cucumber [31].

While the general effects of FR light on plant growth and morphology are clear, and red and FR light can synergistically increase photosynthesis, most FR-light studies delivered a constant red PFD with incremental additions of FR light to decrease the R:FR. It has not yet been established how FR light operates in an environment that has diminishing PFDs of PAR wavebands, such as red light. Furthermore, it is not clear how the substitution of red light for green or the interaction between FR and green light influences lettuce growth and morphology. We grew red- and green-leaf lettuce under lighting conditions where red light was substituted with green light, FR light, or both. Our objectives were to determine: 1) if FR light is as effective as red and/or green light at increasing shoot biomass and 2) how the promotion of growth from FR light depends on other light wavebands. We hypothesized: 1) that the inclusion of FR in the light spectrum would increase biomass accumulation by increasing leaf area and photosynthetic efficiency of red and green light and 2) that the substitution of red light with green light would progressively decrease the promotion of FR light on plant biomass accumulation.

## Materials and methods

### Plant materials and propagation

We sowed 300 seeds of red-leaf ‘Rouxai’ lettuce and green-leaf ‘Rex’ lettuce (Rijk Zwaan USA; Salinas, CA, USA) in a temperature-controlled growth room (Controlled Environment Lighting Laboratory) at Michigan State University (East Lansing, MI, USA). The seeds were sown in 200-cell (2.5 cm × 2.5 cm) rockwool plugs (AO 25/40 Starter Plugs; Grodan, Milton, ON, Canada) that were presoaked in deionized water adjusted to a pH of 4.5 using 10% diluted sulfuric acid (H_2_SO_4_). Lettuce seeds were grown at 23° under a 24 h·d^–1^ photoperiod at a TPFD of 180 μmol∙m^−2^∙s^−1^ from warm-white (peak = 639 nm, correlated color temperature = 2700 K) LEDs until day 3 when the photoperiod was shortened to 20 h·d^–1^. Seedling trays were covered with clear plastic domes from day 0 to 6 to increase humidity. We hand-irrigated seedlings from day 0 to 10 with deionized water supplemented with a water-soluble fertilizer (12N–4P_2_O_5_–16K_2_O RO Hydro FeED; JR Peters, Inc., Allentown, PA, USA) and magnesium sulfate (Epsom salt; Pennington Seed, Inc., Madison, GA) with the following nutrients (in mg∙L^–1^): 125 N, 42 P, 167 K, 73 Ca, 49 Mg, 39 S, 1.7 Fe, 0.52 Mn, 0.56 Zn, 0.13 B, 0.47 Cu, and 0.13 Mo. The pH and the electrical conductivity (EC) were periodically measured by a pH/EC meter (HI9814; Hanna Instruments, Woonsocket, RI, USA) and were 5.6 ± 0.3 and 1.6 ± 0.1 dS∙m^−1^, respectively.

### Growth conditions and lighting treatments

The controlled-environment room consisted of four vertical hydroponic growing racks with three canopies on each rack, allowing us to create twelve independent lighting treatments. On day 10, we separated the seedlings and transplanted 18 of each cultivar into each floating 36-cell raft (Beaver Plastics, Ltd., Acheson, AB, Canada) with 2.5-cm-wide holes that were spaced 20 × 15 cm apart. Plants were provided with the same nutrient solution as previously described but at a 20% higher concentration (i.e., 150 mg N∙L^–1^). We measured the nutrient solution each day and adjusted the pH and EC using potassium bicarbonate and H_2_SO_4_ to maintain an average of 5.7 ± 0.3 and 1.9 ± 0.1 dS∙m^−1^, respectively. During the growth period, we set the air temperature to 23° during the day and night, but the actual air temperature averaged 22.8° during both replications. Plant canopy temperature (24.5 ± 0.5°), relative humidity (39 ± 10%), and CO_2_ concentration (427 ± 22 μmol∙mol^-1^) were also continually measured and were similar during each replication. Additional information about experimental conditions, equipment, and environmental sensors can be found in Kelly et al. [32].

**Fig 1:**
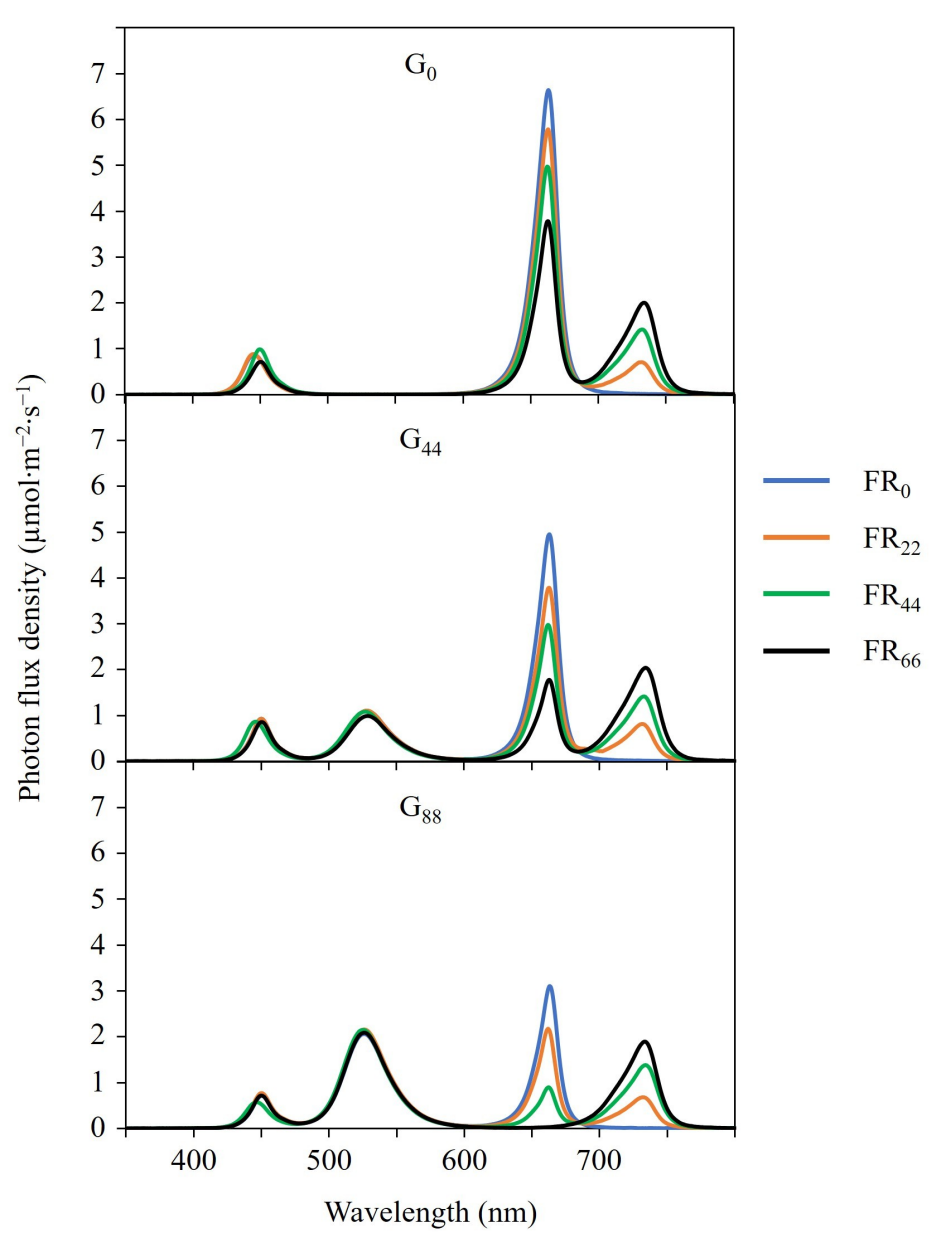
Spectral distributions of lighting treatments. Each treatment had a constant blue (400–499 nm) photon flux density (PFD; μmol∙m^−2^∙s^−1^), a green (G; 500–599 nm) PFD of 0, 44, or 88 μmol∙m^−2^∙s^−1^ (G_0_, G_44_, G_88_), a far-red (FR; 700–799 nm) PFD of 0, 22, 44, or 66 μmol∙m^−2^∙s^−1^ (FR_0_, FR_22_, FR_44_, FR_66_) and a red (R; 600–699 nm) PFD that decreased as the green or FR PFD increased. Each treatment had a target total PFD of 176 μmol∙m^−2^∙s^−1^.

We delivered twelve lighting treatments from day 10 until harvest on days 27 (‘Rex’) and 28 (‘Rouxai’) (Fig 1 and Table 1). Each lighting treatment delivered a TPFD of approximately 176 μmol∙m^−2^∙s^−1^ and a constant blue (peak = 449 nm) PFD of 22 μmol∙m^−2^∙s^−1^. The remaining 154 μmol∙m^−2^∙s^−1^ was delivered by different proportions of light by narrowband green (peak = 526 nm), red (peak = 664 nm), and FR (peak = 733 nm) LEDs. Ninety-five percent of the FR photons had wavelengths between 700 and 750 nm. Three different groups of lighting treatments that delivered green PFDs of 0, 44, or 88 μmol∙m^−2^∙s^−1^ replaced red light, which were designed to determine the effects of an increasing FR PFD in a decreasing red-light environment. The TPFD and spectrum of each lighting treatment were measured at nine locations at plant canopy level using a portable spectroradiometer (PS200; Apogee Instruments, Inc., Logan, UT, USA), and means per treatment are reported.

**Table 1:** Photon flux densities of lighting treatments. Actual individual waveband, total (300–799 nm), and yield photon flux densities [8] delivered in each lighting treatment. Treatments consisted of different combinations of blue (B; 400–499 nm), green (G; 500–599 nm), red (R; 600–699 nm), and far-red (FR; 700–799 nm) light to achieve a target total photon flux density (TPFD) of 176 μmol∙m^−2^∙s^−1^. Yield photon flux density (YPFD) ranged from 99.2 to 160.3 μmol∙m^−2^∙s^−1^. Subscripted values of the lighting treatments denote waveband photon flux densities in μmol∙m^−2^∙s^−1^.

Lighting treatment	Photon flux density (PFD; μmol∙m^−2^∙s^−1^)					
	Blue	Green	Red	Far-red	TPFD	YPFD
B_22_G_0_R_154_FR_0_	21.7	1.0	154.1	1.9	178.7	160.3
B_22_G_0_R_132_FR_22_	21.7	0.9	132.1	22.2	177.2	143.2
B_22_G_0_R_110_FR_44_	22.0	0.7	113.8	45.2	181.8	130.0
B_22_G_0_R_88_FR_66_	20.2	0.6	87.6	66.2	174.7	103.7
B_22_G_44_R_110_FR_0_	22.7	45.7	110.2	1.4	180.3	155.4
B_22_G_44_R_88_FR_22_	22.7	46.1	87.3	24.6	180.6	136.6
B_22_G_44_R_66_FR_44_	23.2	44.2	67.5	45.0	180.1	121.7
B_22_G_44_R_44_FR_66_	21.2	41.6	43.5	68.8	175.3	99.2
B_22_G_88_R_66_FR_0_	21.3	86.1	67.8	1.0	176.5	146.2
B_22_G_88_R_44_FR_22_	22.8	90.3	47.7	21.1	182.2	135.3
B_22_G_88_R_22_FR_44_	20.2	88.7	22.7	44.7	176.6	111.9
B_22_G_88_R_0_FR_66_	21.3	86.7	6.9	61.8	177.0	99.4

### Data collection and analysis

Before destructive plant measurements on days 27 (‘Rex’) and 28 (‘Rouxai’), we measured the relative chlorophyll concentration (SPAD) of eight randomly selected plants of each cultivar from each floating raft by measuring three spots on one fully expanded leaf exposed to direct light using a SPAD meter (MC-100; Apogee Instruments, Inc, Logan, UT) and averaged them. We measured the leaf coloration of lettuce ‘Rouxai’ by taking overhead pictures of three representative plants of each cultivar from each treatment and analyzed them using an R code developed to determine the lightness (black: L* = 0; white: L* = 100), redness (green: a* = −128; red: a* = 127), and blueness (blue: b* = −128; yellow: b* = 127) of each pixel of an imported TIFF image. The L*, a*, and b* values of each pixel were generated and averaged to quantify the average coloration of an entire plant from overhead.

On day 27 or 28, we collected destructive morphological data from eight randomly selected plants of each cultivar (avoiding those on the raft edge) from each floating raft under each lighting treatment. We measured lettuce shoot mass using an analytical balance (AG245; Mettler Toledo, Columbus, OH, USA) and separated the fifth fully expanded leaf. We measured plant diameter (cm), leaf number (> 2 cm in length), and leaf area of the fifth fully expanded leaf (cm^2^). Lettuce shoots and the fifth fully expanded leaf were then put into separate paper bags to be dried for six days in a drying oven (Blue M, Blue Island, IL, USA). After the lettuce shoots and separate leaves were dried, the dry mass (DM) of the fifth fully expanded leaf was measured as well as all dry shoot tissue. Finally, we calculated the specific leaf area of the fifth fully expanded leaf (cm^2^∙g^–1^).

We conducted the experiment as a randomized complete block design (RCBD) with two replications in time, treating each replication as a block, each light treatment as an experimental unit, and each plant within a treatment as an observation unit. The data from both replications were pooled and analyzed using R statistical software [33]. Multiple linear regression and analysis of variance (ANOVA) were used to assess the effects of substituting FR light for green or red light, with the regression model including the PFD of FR light, green light, and their interaction. All data points (2 replications × 12 treatments × 8 observation units = 192) were included in the model. ANOVA with light treatment as the main factor was also performed, followed by Tukey’s honestly significant difference (HSD) test (α = 0.05) to identify statistical differences between light treatments. Data analysis was carried out using the ’dplyr’ [34] and ’agricolae’ [35] packages in R.

## Results

### Leaf characteristics

**Fig 2:**
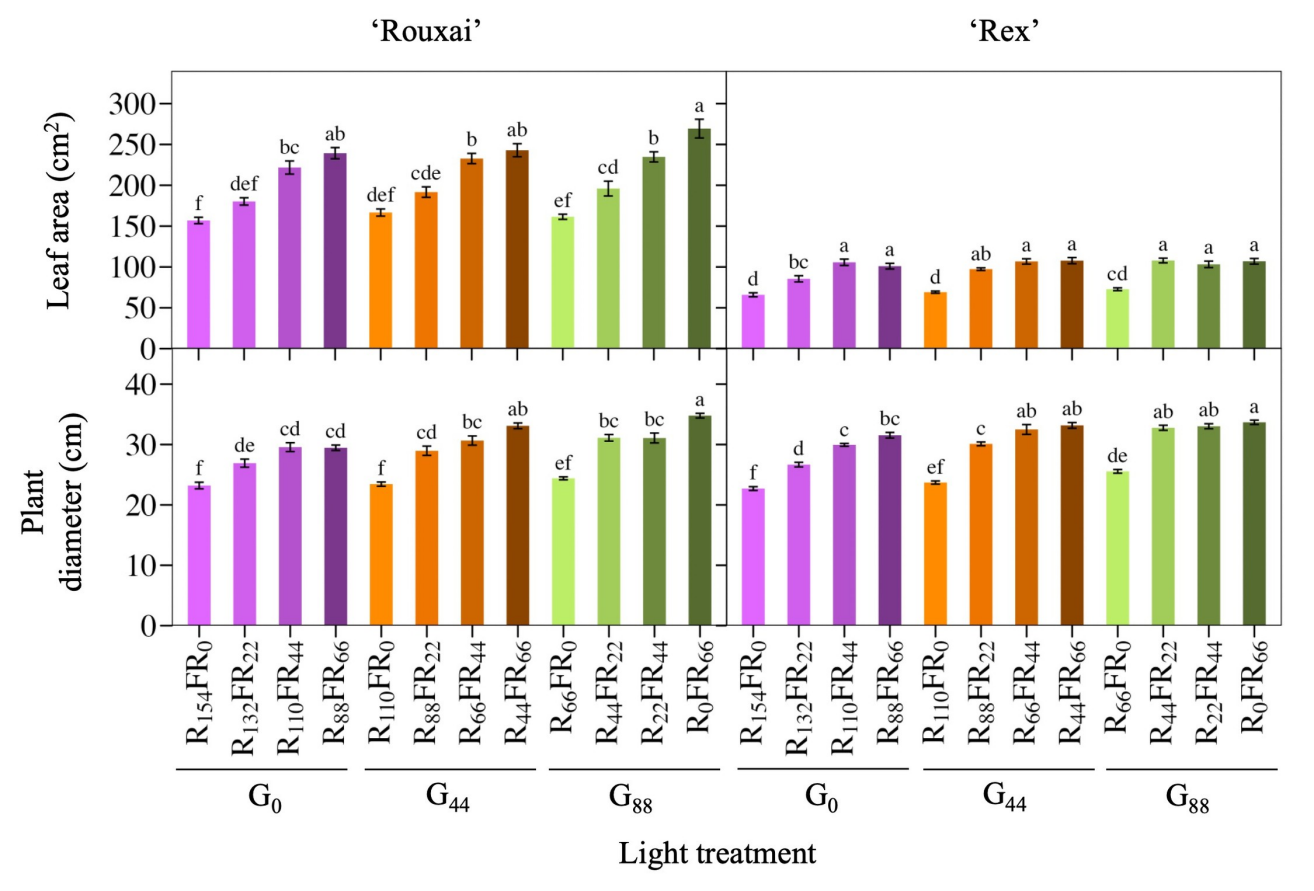
Leaf area and plant diameter. Mean leaf area of the fifth fully expanded leaf and plant diameter of lettuce ‘Rouxai’ and ‘Rex’. Plants were grown under the same total and blue photon flux density (PFD) but different PFDs of green (G; 500–599 nm), red (R; 600–699 nm), and far-red (FR; 700–799 nm) light. Subscript values indicate the PFD of each waveband, in μmol∙m^−2^∙s^−1^. Each bar represents the mean of two replications with eight biological samples per treatment and replication (n = 16). Means with different letters are significantly different based on Tukey’s honestly significant difference test (α = 0.05). Error bars indicate the standard error of each treatment.

In general, the FR PFD {and thus the R:FR and FR fraction [FR/(R+FR)]} had greater effects on lettuce growth attributes than the green PFD (Table 2), although since the TPFD was kept constant, increasing the PFD of one waveband meant another was decreased. Substituting red with FR light increased the leaf area and plant diameter of both cultivars, while substituting red with green light only slightly increased those metrics (Fig 2). For example, at a green PFD of 88 μmol∙m^−2^∙s^−1^, increasing the FR PFD from 0 to 66 μmol∙m^−2^∙s^−1^ (with a corresponding decrease in the red PFD) increased leaf area of ‘Rouxai’ and ‘Rex’ by 66 and 47%, respectively. When green light was almost completely replaced by red light, increasing the PFD of FR light from 0 to 66 μmol∙m^−2^∙s^−1^ increased ‘Rouxai’ and ‘Rex’ leaf area by 52 and 53%, respectively. At the same FR PFD, green light did not affect the leaf area of either cultivar, except for a slight increase in ‘Rex’ leaf area at an FR PFD of 22 μmol∙m^−2^∙s^−1^ when the green PFD increased from 0 to 88 μmol∙m^−2^∙s^−1^. The plant diameter of both cultivars followed similar trends to leaf area (Fig 2). For instance, increasing the FR PFD from 0 to 66 μmol∙m^−2^∙s^−1^ greatly increased plant diameter, regardless of the green PFD, while increases in the green PFD had a minor effect. However, at an FR PFD of 22 μmol∙m^−2^∙s^−1^, increasing the green PFD from 0 to 88 μmol∙m^−2^∙s^−1^ increased the plant diameter of ‘Rouxai’ by 16% and slightly more for ‘Rex’ (23%).

**Table 2:** Two-factor analysis of variance.

	‘Rouxai’			‘Rex’		
Factor	G	FR	G × FR	G	FR	G × FR
Fresh mass	0.101	<0.001	0.809	0.301	<0.001	0.129
Dry mass (DM)	0.171	0.025	0.033	0.317	<0.001	0.030
DM, fifth leaf	0.732	<0.001	0.499	0.706	<0.001	0.794
Area, fifth leaf	0.005	<0.001	0.369	<0.001	<0.001	0.010
Specific leaf area, fifth leaf	<0.001	<0.001	0.318	0.528	0.020	0.024
Plant diameter	<0.001	<0.001	<0.001	<0.001	<0.001	<0.001
Leaf number	0.004	<0.001	<0.001	0.341	<0.001	0.078
SPAD	<0.001	<0.001	0.795	0.001	<0.001	0.057
L*	0.052	<0.001	0.583	–	–	–
a*	<0.001	<0.001	0.038	–	–	–
b*	<0.001	<0.001	0.413	–	–	–

*P* values indicate the main effects of the green (G) photon flux density, far-red (FR) photon flux density, or their interaction on plant biometrics including relative chlorophyll content (SPAD), L* (lightness), a* (redness), and b* (blueness) of lettuce ‘Rouxai’ and ‘Rex’.– = not measured.

Lettuce leaf number, on the other hand, decreased as the FR PFD increased, while green light had a minimal effect. As leaf expansion and plant diameter of both cultivars increased with increases in FR light, leaf number decreased (Table 3). For example, at a green PFD of 88 μmol∙m^−2^∙s^−1^, lettuce grown under 66 μmol∙m^−2^∙s^−1^ of FR light had approximately 3 fewer leaves than those grown without FR light (Table 3). Finally, lettuce that was grown under a higher FR light percentage had a higher specific leaf area (cm^2^∙g^–1^), indicating that there was more leaf surface area for every gram of biomass (Table 3). Therefore, the leaves likely became thinner as the FR light replaced red light.

**Table 3:** Lettuce leaf characteristics. Mean leaf number, dry mass (DM) of the fifth fully expanded leaf (g), and specific leaf area (SLA; cm^2^∙g^–1^) of the fifth fully expanded leaf of ‘Rouxai’ and ‘Rex’. Treatment subscripts indicate the photon flux density (μmol∙m^−2^∙s^−1^) of blue (B; 400–499 nm), green (G; 500–599 nm), red (R; 600–699 nm), and far-red (FR; 700–799 nm) light. Each value represents the mean of two replications with eight biological samples per treatment and replication. Means with different letters are significantly different based on Tukey’s honestly significant difference test (α = 0.05).

Cultivar	Treatment	Leaf number	Dry mass fifth leaf	SLA fifth leaf
Rouxai	B_22_G_0_R_154_FR_0_	13.4 abc	0.217 bcd	744 d
	B_22_G_0_R_132_FR_22_	14.8 a	0.217 bcd	833 bcd
	B_22_G_0_R_110_FR_44_	13.8 ab	0.247 abc	927 abc
	B_22_G_0_R_88_FR_66_	12.0 d	0.270 a	890 abcd
	B_22_G_44_R_110_FR_0_	13.3 bcd	0.219 abcd	766 cd
	B_22_G_44_R_88_FR_22_	14.1 ab	0.221 abcd	875 abcd
	B_22_G_44_R_66_FR_44_	12.9 bcd	0.263 abc	899 abcd
	B_22_G_44_R_44_FR_66_	12.3 cd	0.252 abc	975 ab
	B_22_G_88_R_66_FR_0_	13.9 ab	0.211 cd	785 cd
	B_22_G_88_R_44_FR_22_	13.9 ab	0.195 d	1020 a
	B_22_G_88_R_22_FR_44_	13.1 bcd	0.258 abc	987 ab
	B_22_G_88_R_0_FR_66_	10.4 e	0.268 ab	1019 a
Rex	B_22_G_0_R_154_FR_0_	14.6 ab	0.106 bc	648 b
	B_22_G_0_R_132_FR_22_	14.9 ab	0.122 abc	712 ab
	B_22_G_0_R_110_FR_44_	13.8 ab	0.156 a	718 ab
	B_22_G_0_R_88_FR_66_	13.2 bc	0.126 abc	914 a
	B_22_G_44_R_110_FR_0_	14.8 ab	0.105 bc	752 ab
	B_22_G_44_R_88_FR_22_	15.3 a	0.130 abc	869 ab
	B_22_G_44_R_66_FR_44_	13.8 ab	0.155 a	718 ab
	B_22_G_44_R_44_FR_66_	13.4 bc	0.141 ab	795 ab
	B_22_G_88_R_66_FR_0_	14.8 ab	0.096 c	805 ab
	B_22_G_88_R_44_FR_22_	15.4 a	0.121 abc	918 a
	B_22_G_88_R_22_FR_44_	13.8 ab	0.159 a	697 ab
	B_22_G_88_R_0_FR_66_	11.9 c	0.146 ab	741 ab

### Biomass accumulation

**Fig 3:**
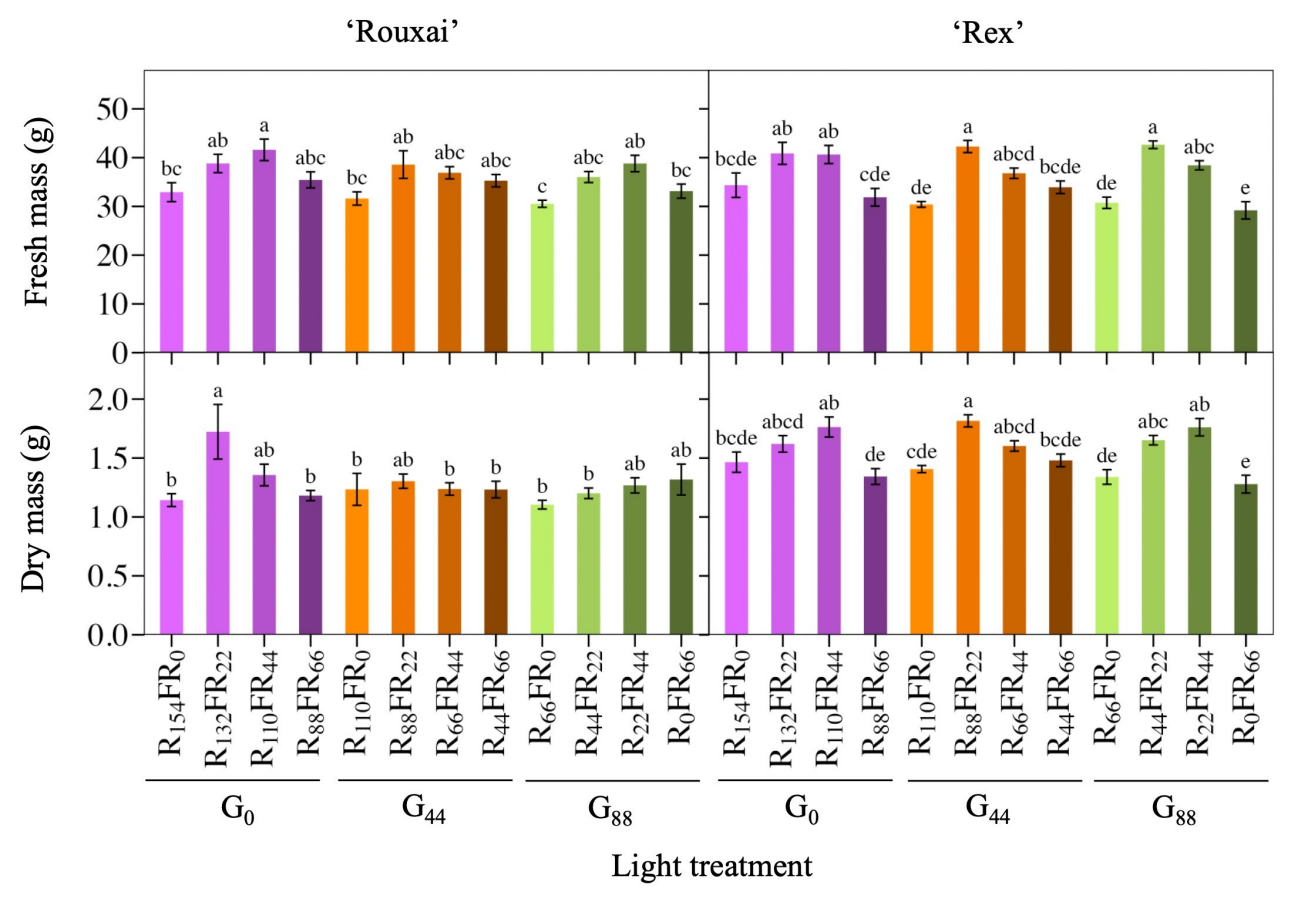
Mean fresh and dry mass of lettuce ‘Rouxai’ ‘Rex’. Plants were grown under the same total and blue (B; 400–499 nm) photon flux density (PFD) but different PFDs of green (G; 500–599 nm), red (R; 600–699 nm), and far-red (FR; 700–799 nm) light. Subscript values indicate the PFD of each waveband, in μmol∙m^−2^∙s^−1^. Each bar represents the mean of two replications with eight biological samples per treatment and replication (n = 16). Means with different letters are significantly different based on Tukey’s honestly significant difference test (α = 0.05). Error bars indicate the standard error of each treatment.

Increasing the FR PFD while proportionately decreasing the red PFD increased lettuce shoot FM, but only until 22 or 44 μmol∙m^−2^∙s^−1^ of FR light replaced red light (Fig 3). Further increases to 66 μmol∙m^−2^∙s^−1^ of FR light led to lettuce with a similar FM as those grown without FR light. For example, FM of both cultivars increased when 22 μmol∙m^−2^∙s^−1^ of FR light replaced red light, except for ‘Rouxai’ when the green PFD was 0 or 44 μmol∙m^−2^∙s^−1^. At 44 μmol∙m^−2^∙s^−1^ of FR light, FM was generally similar to lettuce grown with 22 μmol∙m^−2^∙s^−1^ of FR light. Therefore, at the same TPFD, lettuce grown with 0 or 66 μmol∙m^−2^∙s^−1^ of FR light generally had similar and less FM than those grown with 22 or 44 μmol∙m^−2^∙s^−1^ of FR light. DM of ‘Rex’ followed similar trends to FM, while the DM of ‘Rouxai’ was similar among most treatments.

Green light had a less-pronounced effect on the FM and DM of both cultivars. At any given FR PFD (0, 22, 44, or 66 μmol∙m^−2^∙s^−1^), increasing the green PFD from 0 to 44 or 88 μmol∙m^−2^∙s^−1^ did not increase FM of either cultivar. However, green light did slightly influence the effect of FR light on FM. For example, without green light, increasing the FR PFD from 0 to 22 μmol∙m^−2^∙s^−1^ did not increase ‘Rex’ FM, but at a green PFD of 44 of 88 μmol∙m^−2^∙s^−1^, there was a significant increase. However, this trend was not uniform among cultivars. DM on the other hand, was slightly more affected by green light’s interaction with FR light. ‘Rouxai’ DM increased from a FR PFD of 0 to 22 μmol∙m^−2^∙s^−1^, but only in the absence of green light. ‘Rex’ DM followed similar trends to FM.

### Leaf coloration and chlorophyll concentration

**Fig 4:**
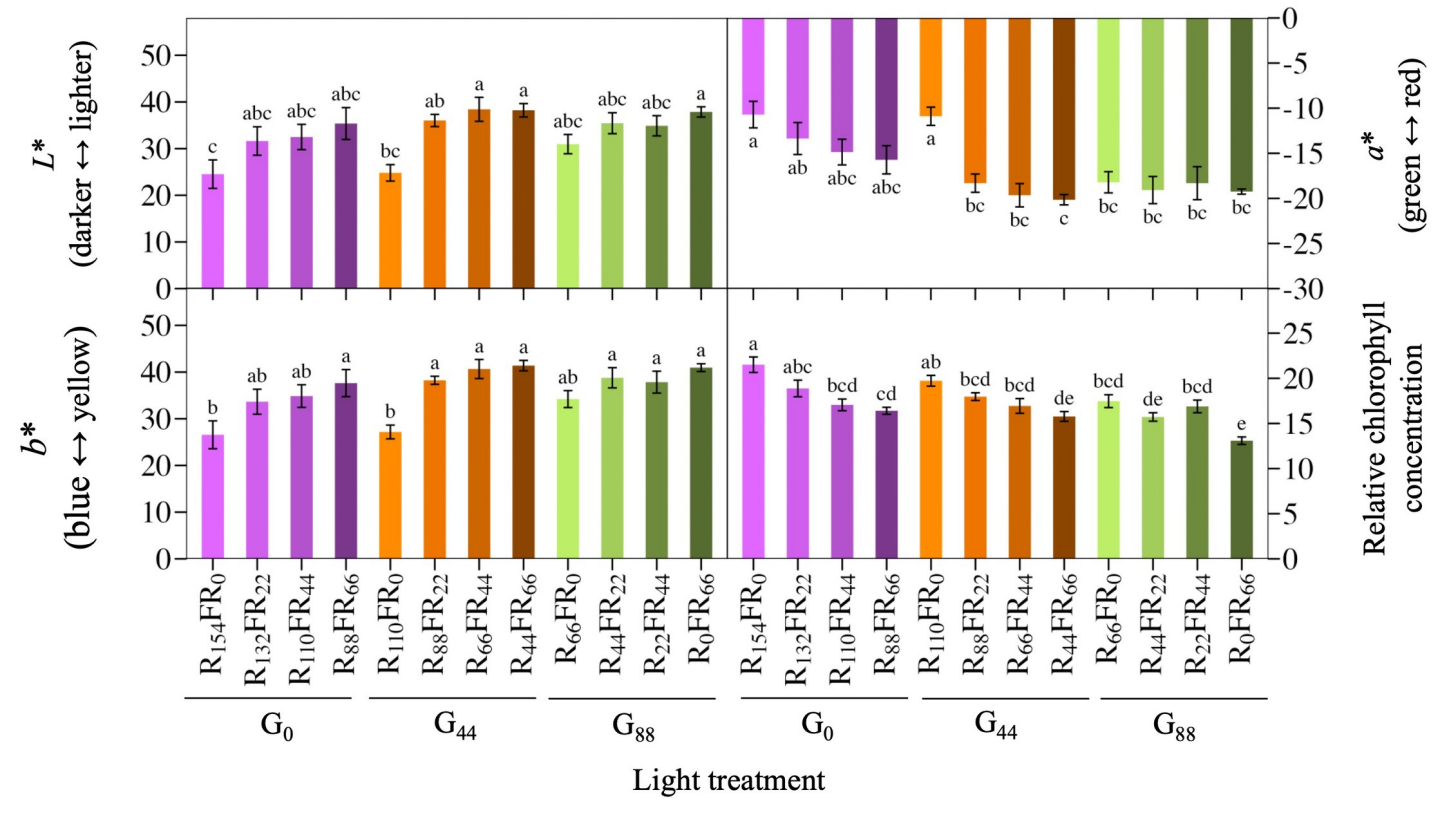
Lettuce coloration index. Mean leaf pigmentation indicated by L*a*b* coloration index values and mean relative chlorophyll concentration (SPAD) of lettuce ‘Rouxai’. Plants were grown under the same total and blue photon flux density (PFD) but different PFDs of green (G; 500–599 nm), red (R; 600–699 nm), and far-red (FR; 700–799 nm) light. Subscript values indicate the PFD of each waveband, in μmol∙m^−2^∙s^−1^. Each bar represents the mean of two replications with three biological samples per treatment and replication, except for SPAD (n = 16). Means with different letters are significantly different based on Tukey’s honestly significant difference test (α = 0.05). Error bars indicate the standard error of each treatment.

**Fig 5:**
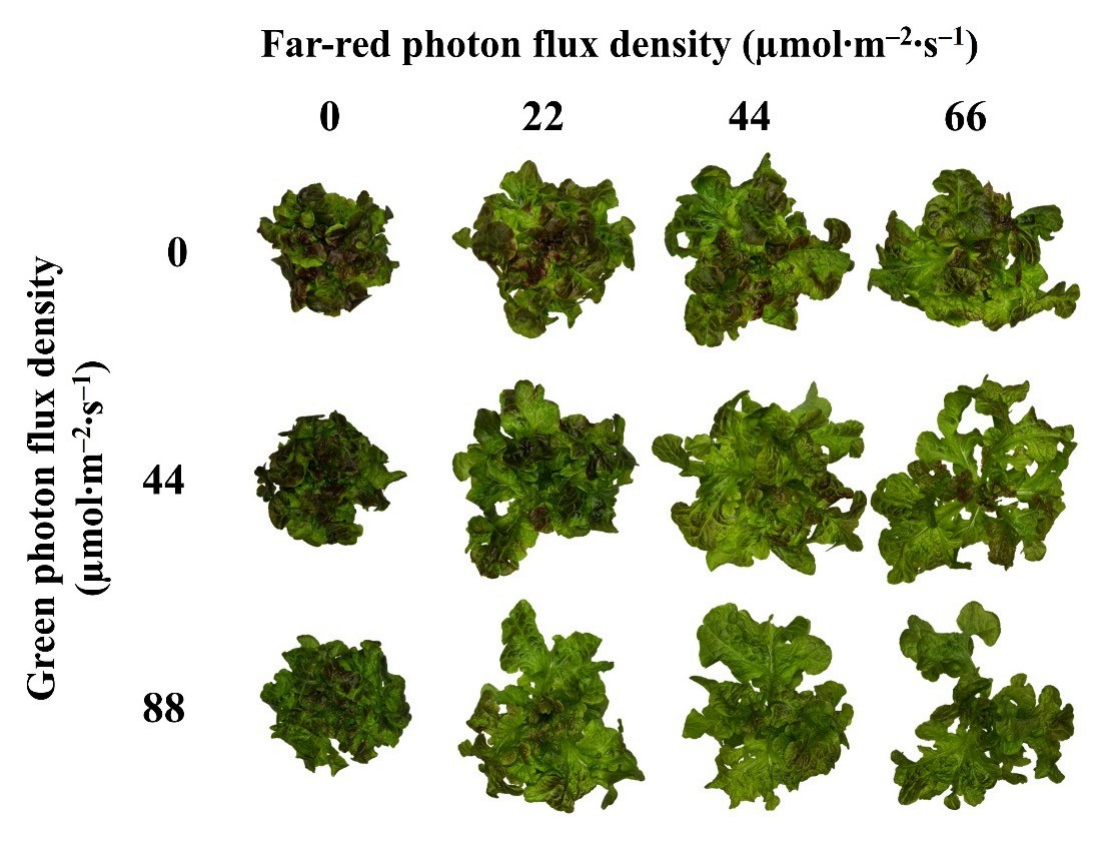
Representative plants from each treatment. Representative plants of lettuce ‘Rouxai’ grown under the same total (300–799 nm) and blue (400–499 nm) photon flux density (PFD; 176 μmol∙m^−2^∙s^−1^ and 22 μmol∙m^−2^∙s^−1^, respectively) but different PFDs of green (500–599 nm), red (600–699 nm), and far-red (700–799 nm) light.

Lettuce ‘Rouxai’ leaf coloration was influenced by the FR and green PFD, although there was an interactive effect on leaf redness (a*) (Table 2). Generally, an increasing substitution of red light with FR light increased leaf lightness (L*), increased yellowness (b*), and decreased redness (a*), especially at the low to moderate green PFDs (Figs 4 and 5). For example, at a green PFD of 44 μmol∙m^−2^∙s^−1^, replacing red with FR light from 0 to 22, 44, or 66 μmol∙m^−2^∙s^−1^ decreased leaf redness by up to 85%. In addition, in the near absence of FR light, increasing the green PFD from 0 or 44 μmol∙m^−2^∙s^−1^ to 88 μmol∙m^−2^∙s^−1^ decreased ‘Rouxai’ leaf redness by about 70%. As lettuce plants became redder, they became bluer (lower b*) and darker (lower L*). The relative chlorophyll concentration (SPAD) of lettuce ‘Rouxai’ decreased as the FR PFD increased from 0 to 66 μmol∙m^−2^∙s^−1^ regardless of the green PFD (Fig 4). Finally, the green PFD had a slight effect on SPAD. When FR light was absent, increasing the green PFD 0 to 88 μmol∙m^−2^∙s^−1^ slightly decreased the SPAD index.

### Predictors of leaf area and biomass accumulation

**Fig 6:**
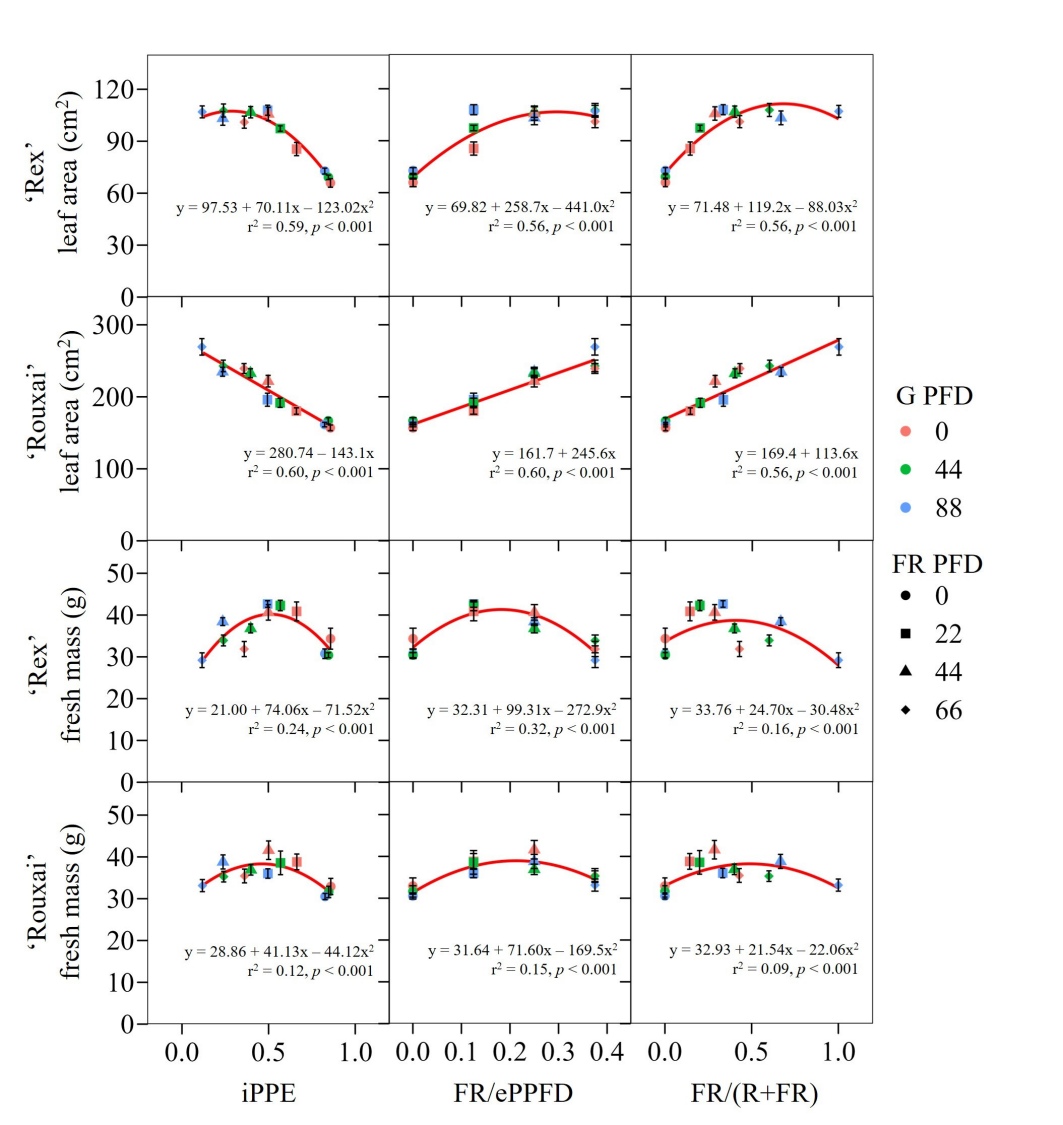
Linear and quadratic relationships of fresh mass and leaf area. Mean fresh mass (whole plant) and leaf area of the fifth fully expanded leaf of lettuce ‘Rex’ and ‘Rouxai’. Each symbol color represents a different green (G, 500–599 nm) photon flux density (PFD) and each shape represents a different far-red (FR; 700–799 nm) PFD. Each symbol represents the mean of two replications with eight biological samples per treatment and replication (n = 16). Error bars indicate the standard error of each treatment. Regression lines, equations, and r^2^, and *p* values were calculated based on linear or quadratic relationships between estimated internal phytochrome photoequilibria (iPPE), the fraction of FR light to the extended photosynthetic PFD (ePPFD; 400–799 nm) (FR/ePPFD), or FR fraction [FR/(red (600–699 nm)+FR)], and raw leaf area data (n = 192).

We explored the relationship of leaf area and FM to the iPPE, the fraction of FR light to the extended PPFD (400–799 nm) (FR/ePPFD), and FR fraction (Fig 6). In general, leaf area increased as the iPPE decreased, although for ‘Rex’ it reached an approximate maximum when the iPPE was <0.5. Leaf area increased as the FR/ePPFD or FR fraction increased, but apparently reached plateaus for ‘Rex’ at around 0.25 and 0.5, respectively. The relationship between FM and each metric was quadratic for both cultivars. FM increased as the iPPE, FR/ePPFD, or FR fraction increased, but then began to decrease beyond intermediate values (e.g., 0.5, 0.25, and 0.4, respectively). There was a slightly stronger correlation for ‘Rex’ compared to ‘Rouxai’.

## Discussion

### Increasing the FR PFD increased leaf expansion and biomass accumulation

Many sole-source lighting studies investigated the addition of FR to a light spectrum, which increased the TPFD. For example, adding FR light to a red+blue light spectrum increased leaf area, regardless of the R:FR, compared to a fluorescent or red+blue light control [23]. Furthermore, the addition of 30 μmol∙m^−2^∙s^−1^ of FR light to a red, blue, or red+blue light spectrum increased lettuce leaf length, but the effect of FR light was more pronounced under a higher blue:red than a lower one [24]. In the same study, adding up to 75 μmol∙m^−2^∙s^−1^ of FR light to 180 or 360 μmol∙m^−2^∙s^−1^ of blue+red (blue:red = 1:1) light increased lettuce leaf length, but to a greater extent when added to the low blue+red PFD. We observed similar increases in leaf expansion when red light was substituted by the same PFD of FR light, which maintained a constant TPFD. In addition, leaf number decreased as leaf area increased under an increasing FR PFD. Previous studies reported similar responses, where leaf number decreased with an increasing FR fraction [24, 36]. This could be attributed to a shift in resource allocation from leaf primordium growth towards leaf and stem elongation due to a shift in phytochrome status caused by prolonged perceived shade conditions [37].

The increase in leaf area and decrease in leaf number caused by an increasing FR PFD is a shade-avoidance response controlled by a lower R:FR that shifts phytochrome from the Pfr to Pr form [9, 38, 39]. Shade-avoidance responses, such as leaf expansion and stem elongation, are induced by a low PPE [40]. When a low R:FR shifts the phyB pool from Pfr to Pr, phyB dissociates from PIF4 and PIF5, which allows PIFs to accumulate in the nucleus and promote the expression of shade-avoidance genes such as auxin biosynthesis, transport, and signaling genes as well as gibberellin synthesis genes [38, 41–44]. Additionally, PIFs interact with enzyme complexes such as CONSTITUTIVELY PHOTOMORPHOGENIC 1 (COP1) and SUPRESSOR OF PHYA (SPA), which targets, ubiquitinates, and degrades phyB as well as photomorphogenesis transcription factors like ELONGATED HYPOCOTYL 5 (HY5) [40, 45–47]. The removal of phyB from the nucleus, either by dissociation from PIF4 and PIF5 or degradation mediated by COP1/SPA, allows for the increased expression of shade-avoidance genes that can increase cell elongation and leaf surface area.

In the current study, the increase in leaf area is partly responsible for greater biomass accumulation under high FR-light fractions, but a continual increase in leaf area did not continually increase biomass accumulation. The increase in biomass accumulation is likely due to increased light interception and canopy photosynthesis [4, 48]. Additionally, delivery of FR with red light preferentially excites PSI and increases photosynthetic rates compared to red or FR light alone [5, 18, 19], which increases biomass accumulation. When the FR PFD increased to 22 or 44 μmol∙m^−2^∙s^−1^, FM and DM increased, although the response slightly varied by cultivar and green PFD. However, although an increasing FR PFD continually increased leaf area, FM was similar under 0 or 66 μmol∙m^−2^∙s^−1^ of FR light (and 66 or 0 μmol∙m^−2^∙s^−1^ of red light, respectively). The decrease in FM relative to leaf area could be explained by a decrease in photosynthetic rates caused by a decrease in red light.

Another way to characterize a light spectrum is by calculating its yield photon flux density (YPFD) which is based on the relative quantum efficiency curve for photosynthesis developed by McCree [8, 17]. The relative quantum efficiency of photons is <0.5 at wavelengths <380 nm and >696 nm and is >0.9 at wavelengths from 558–676 nm. Thus, the YPFDs for the twelve treatments in this study were greatest for treatments with the largest percentages of R light and lowest percentages of FR light (Table 1). However, the FR-deficient treatments with the highest YPFDs (e.g., B_22_G_0_R_154_FR_0_ and B_22_G_44_R_110_FR_0_) led to plants with less biomass than treatments with FR light (and lower YPFDs) because of less leaf expansion and thus light interception, as well as the lack of the promotive effect of applying red light with FR light. Thus, delivering little or no red or FR light can decrease plant growth and yield due to less photon capture and photosynthetic efficiency.

### FR light reduced leaf coloration

The red-leaf coloration of lettuce ‘Rouxai’ decreased as the FR or green PFD increased (Fig 4). Since the blue PFD was constant and leaf redness decreased when the green or FR PFD increased, the change in leaf coloration can be attributed to less red light and the increase in leaf expansion caused by green and especially FR light. These results are consistent with a lettuce study in which red light increased leaf redness when 100 μmol∙m^−2^∙s^−1^ was delivered alone or with blue light at the end of the production cycle [49]. In another study that compared responses under a broad-waveband white LED, increasing the FR PFD and decreasing the R:FR from 11.5 to 0.5 increased leaf expansion, but also decreased anthocyanin content and presumably leaf redness of lettuce [21]. Finally, the addition of FR light to a blue+red spectrum decreased leaf redness at low and moderate PPFDs [24].

Lettuce leaf redness is strongly associated with anthocyanin content [50]. Incorporating FR light into the light spectrum often decreases anthocyanin concentration due to the simultaneous increase in leaf area. As leaves expand at a faster rate from FR light, anthocyanin concentrations become more diluted [51]. For example, adding FR light to white light, at the same TPFD, decreased total anthocyanin concentrations compared to white light alone or white plus blue light [52]. Furthermore, as the FR PFD in a white-light background increased, the leaf area of lettuce ‘Cherokee’ increased while anthocyanin content decreased [15]. A decrease in leaf redness can be caused by a decrease in the R:FR and a decrease in PPFD because both red light and a higher PPFD can induce anthocyanin biosynthesis [21, 23, 53, 54]. In the present study, it is likely that 22 μmol∙m^−2^∙s^−1^ of blue light in all treatments led to similar anthocyanin biosynthesis between treatments, and the partial substitution of R with FR light in the spectrum increased leaf area, thus diluting anthocyanin concentrations and leaf pigmentation.

### Green light and its interaction with FR light

Research has shown that green light can elicit some similar shade-avoidance responses as FR light [25, 30, 55], although in the current study, green light was less effective than FR light at inducing these responses. Furthermore, green and FR could interact to influence shade-avoidance responses, at least at very low total PFDs [56]. In this study, when FR light was absent, and green light replaced red light at the highest PFD tested (B_22_G_88_R_66_FR_0_), FM, DM, leaf area, and plant diameter were similar to lettuce grown under the maximum red PFD (B_22_G_0_R_154_FR_0_). This indicates that green light can replace red light and lead to similar biomass accumulation likely due to greater photosynthetic rates in lower leaves, which is where red and blue light are relatively deficient [27]. However, there were greater increases in leaf area and biomass accumulation when FR light replaced red light compared to green light replacing red light, at the same PFD, indicating that FR light was a more effective driver of whole-plant photosynthesis than green light.

### Growth relationships to phytochrome estimators

We investigated how metrics that estimate phytochrome status influenced the leaf area and FM of both cultivars (Fig 6). The FR fraction ignores the effects of blue and green light (which are relatively poorly absorbed by phytochrome), has an inverse relationship with iPPE, and is a relatively simple way to correlate morphological responses to phytochrome [57]. The FR/ePPFD is conceptually similar to the FR fraction except it compares FR light to the total photon flux between 400 and 800 nm. In the current study, leaf area increased (reaching maximums for the green-leaf cultivar Rex) as the iPPE of the light treatment decreased or the FR/ePPFD or FR fraction increased. All metrics were good predictors of leaf area, although interestingly the relationship was linear for the purple-leaf ‘Rouxai’ quadratic for ‘Rex’. Response trends for FM were quadratic for all metrics and both cultivars but had lower coefficient of determination values. Notably, FM was similar under a spectrum without FR (and high red light) light or high FR (and low red light). This suggests that a light spectrum with an estimated iPPE of ≈0.5, FR/ePPFD of ≈0.4, or FR fraction of ≈0.25 elicits the greatest lettuce biomass at the TPFD tested and when the blue PFD is relatively low.

## Conclusions

In the current study, replacing PAR with FR light, and thus decreasing the R:FR, increased leaf expansion of lettuce ‘Rouxai’ and ‘Rex’ but decreased leaf coloration, relative chlorophyll concentration, SLA, and overall visual appearance. Additionally, when the maximum FR PFD replaced red light (low R:FR), regardless of the green PFD, lettuce FM was similar to a spectrum lacking FR light (high R:FR). This indicates that a continual increase in FR light and leaf expansion did not correlate with greater FM accumulation when the spectrum was deficient in red light, but sufficient in green light. Therefore, the inclusion of some FR in a light spectrum can increase lettuce FM and potentially lower energy input costs for lighting, but red light is necessary to maximize growth because green light was slightly less effective. Indoor growers of leafy-green vegetables can increase biomass accumulation by delivering a modest far-red light fraction (e.g., 10 to 25%), but there can be trade-off effects on crop quality, especially when the green PFD is high.
